# Editorial: Filling the knowledge gap of neonatal hemostasis

**DOI:** 10.3389/fped.2022.1110481

**Published:** 2023-01-04

**Authors:** Rozeta Sokou, Nicoletta Iacovidou, Stavroula Parastatidou, Aikaterini Konstantinidi

**Affiliations:** ^1^ Neonatal Intensive Care Unit, Nikaia General Hospital “Aghios Panteleimon”, Piraeus, Greece; ^2^ Department of Neonatology Aretaieio Hospital, National and Kapodistrian University of Athens, Athens, Greece; ^3^ Neonatal Intensive Care Unit, University General Hospital Attikon, Athens, Greece

**Keywords:** developmental hemostasis, neonatal hemostasis, hematology, coagulopathy, neonates, bleeding events

**Editorial on the Research Topic** Filling the knowledge gap of neonatal hemostasis

“Developmental hemostasis” is a term used to describe the dynamic process of gradual hemostatic maturation from fetal life through the adulthood (1). Neonates are born with a hemostatic “deficit” related to the gestational age, the immaturity of hepatic function, the varying vitamin K concentrations and the developmental stage of vascular endothelium (2).

Despite the continuous advances in neonatal hematology, there remain many gaps in our understanding of the hemostatic mechanisms in this delicate population. The existing significant qualitative and quantitative differences of coagulation factors between neonates and adults make the interpretation of hemostatic alterations in neonates difficult, resulting in pitfalls and dilemmas that do not occur in adults (3). In neonates platelet counts are similar to adults, as it is reported in the literature, yet there are developmental differences with respect to platelet function. It is noteworthy, that despite platelet hyporeactivity in neonates, primary hemostasis is not impaired; bleeding times (BTs) in healthy term neonates as well as closure times (using the Platelet Function Analyzer-PFA-100) are found to be shorter than older children and adults (4, 5).

Conventional coagulation tests such as prothrombin time (PT), international normalized ratio (INR), activated partial thromboplastin time (APTT), although commonly used for the assessment of coagulopathies, present limitations; they do not provide adequate information on platelet function and fibrinolysis (2, 6, 7). Moreover, in neonates the presence of abnormal values of these tests are not always associated with a clinically relevant disease and what is more important, their predictive value for bleeding is poor. On the other hand, viscoelastic tests such as Thromboelastography/Thromboelastometry (TEG/ROTEM), based on the interactions between blood cells and coagulation factors can estimate the dynamics of blood coagulation from the activation of clotting factors to clot lysis. Thus, they may represent a useful tool for studying the coagulation status of neonates (7). Several studies have conducted aiming at establishing reference ranges for the coagulation parameters in neonates but there is no study establishing cut-off values for bleeding risk in this population. This fact has led clinicians to focus on correcting coagulation values outside the reference ranges and to transfuse critically ill neonates without evidence of bleeding (2).

The management of hemostatic derangements in neonates represents a challenge for neonatologists, as neonatal bleeding or thromboembolic events are potentially life-threatening situations calling for accurate diagnostic tests, better markers for treatment decision making, and also for a new therapeutic approach.

The Research Topic “*Filling the knowledge gap of neonatal hemostasis*” includes four novel and original contributions in the field of neonatal hemostasis aiming at highlighting recent advances on pathophysiology, diagnosis and prediction of hemostatic derangements in sick neonates and providing new insights in the management of specific clinical settings.

In this collection of articles, Amelio et al. investigate the hemostatic profile of 26 full term and 19 late preterm neonates hospitalized in a Neonatal Intensive Care Unit (NICU) during the first 72 h of life, using a novel device named Viscoelastic Coagulation Monitor (VCM®); they studied the relationship between the values of VCM parameters and conventional coagulation tests. Additionally, they evaluate the differences between the values of VCM parameters found in their cohort with relevant adult reference values displayed by the same device.

In healthy neonates, the hemostatic system although immature is functionally balanced, yet this balance may easily be disturbed in the context of critical illness such as sepsis, birth asphyxia, major surgery, liver disease, extracorporeal membrane support (ECMO) resulting in bleeding or thromboembolic events (8).

Cortesi et al. provide an overview of different monitoring tests and approaches, with focus on the role of point-of-care (POC) viscoelastic assays in neonatal ECMO. The authors come to the conclusion that the best strategy to monitor and guide anticoagulation therapy is still unknown; a combination of multiple laboratory assays appears to be the best option for the assessment of the coagulation status in neonates undergoing ECMO.

Regarding platelet functionality of neonates with fetal growth restriction (FGR), Kollia et al. evaluate platelet responsiveness using PFA-100 in cord blood samples and report that FGR neonates demonstrate a relatively hyporesponsive platelet phenotype, especially those who are preterm, compared to healthy neonates.

Inflammation and hemostasis are interrelated pathophysiologic processes that considerably affect each other. In this two-way relationship, inflammation promotes coagulation activation which in turn influences inflammatory activity. The bi-directional relationship between coagulation and inflammation appears to play a key role in the process underling multi-organ dysfunction in septic patients (9).

In the context of this relationship, Sokou et al. develop and validate a neonatal sepsis diagnostic (NeoSeD) model for hospitalized neonates undergoing evaluation for sepsis, combining ROTEM parameters and maternal/neonatal risk factors, clinical and laboratory variables. Authors conclude that this score may contribute to the timely diagnosis, optimal management, and potential improvement of short-and long-term outcome of septic neonates.

Neonatal hemostasis is a serious issue not adequately studied due to difficulties attributed to very nature of the neonatal population. Thorough comprehension of the developmental stage of hemostasis and the underlying pathophysiology is considered as a prerequisite for the appropriate evaluation of risk factors and proper management of hemostatic derangements in neonatal population.

This Research Topic attempts to illuminate the field of neonatal hemostasis and provide interesting and documented data as well as stimuli for further research. However, there remains an urgent need for filling the gaps in knowledge of neonatal transfusion therapy along with the establishment of international collaboration to develop evidence-based guidelines.
